# Multi-Parametric Analysis of Below-Knee Compression Garments on Delayed-Onset Muscle Soreness

**DOI:** 10.3390/ijerph18073798

**Published:** 2021-04-06

**Authors:** Thilo Hotfiel, Svenja Höger, Armin M. Nagel, Michael Uder, Wolfgang Kemmler, Raimund Forst, Martin Engelhardt, Casper Grim, Rafael Heiss

**Affiliations:** 1 Department of Orthopedic Surgery, Friedrich-Alexander-Universität (FAU) Erlangen-Nürnberg, Rathsberger Str. 57, 91054 Erlangen, Germany; Thilo.Hotfiel@klinikum-os.de (T.H.); Raimund.Forst@fau.de (R.F.); 2 Center for Musculoskeletal Surgery Osnabrück (OZMC), Klinikum Osnabrück, Am Finkenhügel 1, 49076 Osnabrück, Germany; Martin.Engelhardt@klinikum-os.de (M.E.); casper.grim@klinikum-os.de (C.G.); 3 Institute of Radiology, University Hospital Erlangen, Friedrich-Alexander-Universität (FAU) Erlangen-Nürnberg, Maximiliansplatz 3, 91054 Erlangen, Germany; SvenjaHoeger@gmail.com (S.H.); armin.nagel@uk-erlangen.de (A.M.N.); michael.uder@uk-erlangen.de (M.U.); 4 Institute of Medical Physics (IMP), Friedrich-Alexander-Universität (FAU) Erlangen-Nürnberg, Henekstr. 91, 91052 Erlangen, Germany; Wolfgang.Kemmler@imp.uni-erlangen.de

**Keywords:** ergogenic aids, sports injuries, muscle injuries, muscle fatigue, regeneration, recovery

## Abstract

To investigate below-knee compression garments during exercise and a post-exercise period of 6 h on clinical, functional, and morphological outcomes in delayed-onset muscle soreness (DOMS). Eighteen volunteers (age: 24.1 ± 3.6, BMI 22.7 ± 2.7 kg/m^2^) were enrolled. Measures were acquired at baseline, 6 h, and 48 h after eccentric and plyometric exercise, with wearing a compression garment (21–22 mmHg) on a calf during and for the first 6 h after exercise. 3T MRI was performed for quantification of intramuscular edema (T2 signal intensity (SI), T2 time, and manual volume segmentation); jump height, calf circumference, ankle dorsiflexion (DF), creatine kinase (CK), and muscle soreness were assessed. DOMS was confirmed in all participants after 48 h, with an increase in soreness (*p* < 0.001) and CK (*p* = 0.001), decrease in jump height (*p* < 0.01), and the presence of intramuscular edema (*p* < 0.01) in both the compressed and non-compressed limbs. No differences between the compressed and non-compressed limbs were observed for muscle soreness and jump height. MRI T2 SI, T2 time, soreness, and manual segmentation revealed no effect of the compression treatment. The assessment of calf circumference and DF showed no changes in either the compression or non-compression limb (*p* = 1.0). Wearing compression garments during combined eccentric and plyometric exercise and for 6 h post-exercise has no effect on clinical signs of DOMS, jump performance, or the development of intramuscular edema.

## 1. Introduction

High-performance sports and the importance of achieving peak physical capacity and performance have led athletes and coaches to continually seek treatments or techniques that may improve regeneration and recovery [1,2,3]. The rate and quality of recovery has been considered to be essential for both elite and recreational athletes during repetitive training or competition [1,2,3,4].

A wide variety of interventions aiming to either prevent or relieve the development of muscle soreness, thereby accelerating recovery from performance-limiting conditions, have been reported [2]. Compression garments are traditionally applied for vascular purposes (e.g., deep venous and lymphatic disease) or surgery, and their clinical benefits are well documented [5,6,7]. Recently, compression garments have increasingly been used by those participating in sporting activities [6,8,9]. Existing systematic reviews have concluded that compression garments are effective in enhancing recovery from overexertion-related muscle injuries (primarily within 48 h of injury), whereas the majority of previous studies failed to support performance-enhancing effects [1,8,9,10,11,12]. Various mechanisms have been hypothesized to explain the potential beneficial effects of compression garments, including altered muscle biomechanics, leading to reduced power expenditure and microtrauma; improvements in proprioception; elevated tissue pressure; and even placebo effects [8,13]. A limitation of most existing studies is that they have focused on a variety of exercise types, outcome parameters, or types of compression, including different wearing time. This leads to a broad heterogeneity of results and a paucity of data on fundamental physiological mechanisms occurring upon the application of compression therapy for muscular issues [12]. However, identifying the mechanisms, metabolic conditions, and application modes under which compression may alter muscle metabolism and improve recovery is crucial. Hence, there is a need for a highly standardized multiparametric approach to assess clinical, functional, and morphological changes in exercise induced muscle damage such as delayed-onset muscle soreness (DOMS). DOMS is one of the most common reasons for impaired muscle performance in sports and it is associated with muscle soreness, reduced muscle strength, and limited range of motion, and is frequently observed in both professional and recreational athletes [1]. The results might be of high clinical relevance for the understanding of compression principles, the optimization of its application, and the development of further therapeutic regimens. We hypothesized that the use of compression garments (21–22 mmHg) during exercise und the immediate post-exercise phase are able to reduce intramuscular edema and microstructural damage and thereby improve functional performance and recovery in cases of DOMS. The purpose of our study was to investigate the effects of wearing below-knee compression garments during and for 6 h after a standardized plyometric and eccentric exhausting exercise using clinical, functional, and morphological outcome measurements.

## 2. Results

All recruited volunteers successfully performed the plyometric and eccentric exercises described and completed the measures strictly according to the study protocol.

The mean CK value was 174.5 ± 131.7 U/L at baseline and 4423 ± 7489.3 U/L 48 h after the exercise (*p* = 0.001). Moreover, 48 h after the exercise, all subjects presented grade I lesions in the MGM according to the modified Peetrons classification [14,15], indicating the induction of DOMS [16]. Therefore, all 18 volunteers were included in further evaluation.

Muscle soreness significantly increased from baseline (T0) to 48 h follow-up (T2) in both the compressed (0 ± 0 versus 4.7 ± 2.3, *p* < 0.001) and non-compressed (0 ± 0 versus 4.8 ± 2.0, *p* < 0.001) limbs when climbing stairs, whereas no differences were observed at 6 h (T1). No difference in muscle soreness was observed between the compressed and non-compressed limbs at either follow-up time (T1 and T2).

**Figure 1:**
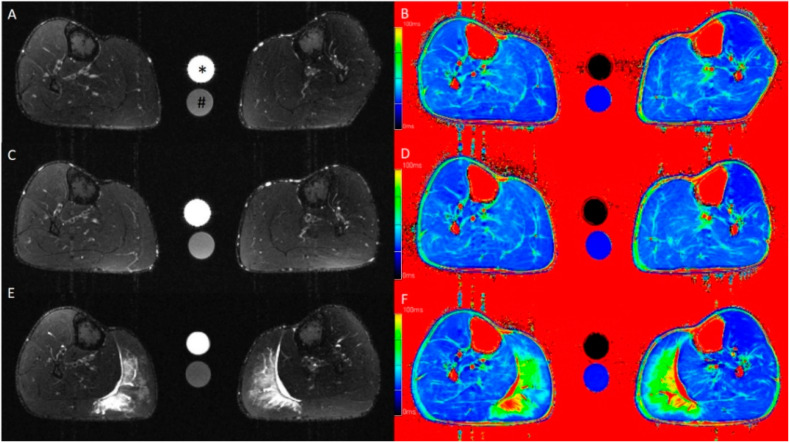
Axial T2-weighted turbo inversion recovery magnitude (**A**,**C**,**E**) and axial T2 mapping (**B**,**D**,**F**) images of the lower leg at baseline (**A**,**B**) and at 6 h (**C**,**D**) and 48 h (**E**,**F**) after standardized exercise of the calf muscles. Muscle edema is apparent in the medial gastrocnemius muscle in the post-exercise images after 48 h. This participant wore the compression garment on the left calf for 6 h after exercise. The calibration tubes contain 40 mmol/L NaCl (*) and 40 mmol/L NaCl with 5% agarose (#).

**Figure 2:**
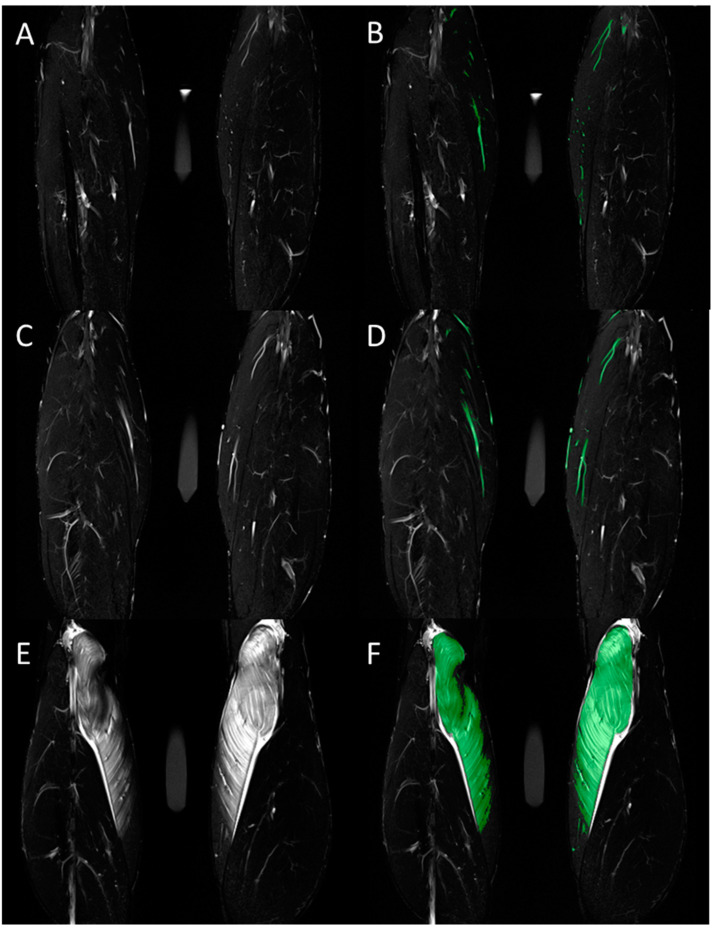
Total volume of intramuscular edema was assessed in the coronal T2-weighted TIRM images. Representative images before (**A**,**C**,**E**) and after (**B**,**D**,**F**) color-coded manual segmentation of the edematous medial gastrocnemius muscle are shown for measurements at baseline (**A**,**B**) and at 6 h (**C**,**D**) and 48 h (**E**,**F**) after standardized exercise. Hyperintense vessels were segmented in the baseline images and subtracted from the segmented volume in the post-exercise images.

Similarly, for jump height, the compression treatment had no statistically significant effect at any time point (*p* = 1.00). A significant reduction in jump height appeared for both the compressed (8.5 ± 2.2 cm versus 6.6 ± 1.7 cm, *p* = 0.008) and non-compressed (8.1 ± 2.0 cm versus 6.8 ± 2.2 cm, *p* = 0.003) leg at T2. The assessment of calf circumference and range of motion showed no statistically significant changes at any time point, nor did wearing the compression garment (*p* = 1.0). However, both calf circumference and range of motion showed a tendency to increase, respectively to decrease, over time. In contrast, T2 SI and T2 time, representing intramuscular edema, demonstrated a significant increase in the MGM in both the compressed (T2 SI: 28.5 ± 13.1 versus 83.1 ± 70.2, *p* = 0.005; T2 time: 36.3 ± 3.9 ms versus 57.7 ± 27.9 ms, *p* < 0.001) and non-compressed (T2 SI: 30.5 ± 10.2 versus 76.2 ± 49.3, *p* = 0.011; T2 time: 36.3 ± 3.3 versus 54.9 ± 23.8, *p* = 0.003) lower leg after 48 h (T2). No effect of wearing a compression garment appeared for T2 SI (*p* = 1.0) or T2 time (*p* = 1.0) of the MGM (Figure 1). Comparable results were achieved using manual segmentation of the volume of total intramuscular edema in the MGM, which showed a significant increase for both the compressed (*p* = 0.025) and non-compressed (*p* = 0.003) legs at T2, with wearing a compression garment having no impact (*p* = 1.0; Figure 2). No gender specific changes occurred for muscle soreness, T2 SI and T2 time, or for the relative changes of calf circumference, jump height, intramuscular edema, and range of motion. Additional results are presented in Table 1.

## 3. Discussion

To the best of our knowledge, this study is the first to investigate the effects of wearing below-knee compression garments (continuously during exercise and for the 6 h post-exercise) on both morphological and functional changes in skeletal muscles by means of a multiparametric analysis.

The underlying approach of using an exercise-induced DOMS model indicating ultrastructural muscle injuries has been successfully established in previous studies [17,18,19,20] and was performed here with eccentric and plyometric exercises. The protocol used in this study can be considered effective, as we were able to observe significant increases in CK levels, muscle soreness, and intramuscular edema in all participants, indicating the development of clinical signs of DOMS after 48 h. DOMS, an ultrastructural muscle injury, is classified as an overexertion muscle disorder [21]. It is one of the most common reasons for impaired muscle performance in sports and is associated with muscle soreness, reduced strength, and range of motion, and is frequently observed in both professional and recreational athletes [16,22,23,24].

The major finding of our study was that the continuous application of compression during combined plyometric and eccentric exercises and throughout a post-exercise period of 6 h showed no influence on muscle edema, soreness, or jump height for all time-points of measurement (at 6 h and 48 h after exercise). These results contradict hypothesis, which predicted altered morphological and functional changes in muscles subjected to compression treatment. The understanding of how and under which conditions muscle tissue responds to compression is essential knowledge for improved recovery and prevention. Several exercise-, athlete-, and compression-related aspects must be contemplated.

DOMS was previously considered to be the result of exhausting or prolonged muscle activity, particularly after eccentric exercise and/or unfamiliar and not-well-coordinated sporting activities [16]. DOMS is associated with ultrastructural damage to the skeletal muscle tissue, involving protein degradation [25]; loss of myofibrillar integrity, with Z-band streaming and disruption; inflammatory processes [26]; and changes in microcirculation and vascular permeability [27].

A recent meta-analysis demonstrated a significant effect of exercise modality on the effects of compression at all time points [28]. The same authors found compression to be most effective for improving recovery from exercise that elicits a large degree of muscle damage [28]; just as demonstrated in our study. Recovery from resistance or plyometric exercise has been demonstrated to derive the greatest benefits from compression [28,29]. Indeed, the wide range in exercise designs, including differences in timing, contraction forms, and intensity, has contributed to apparently inconsistent findings; thus, specific recommendations for the ideal exercise regime supporting the use of compression have not yet been elucidated [11]. The protocol applied in this study consists of an exhausting exercise protocol leading to DOMS. However, no significant effects could be observed for compression on either the functional or morphological level. In this study, plyometric forms of contractions have been included in order to target high-speed plyometric contractions. Notably, in sports, there are no isolated heavy loaded eccentric contractions that induce a “pure eccentric overload” as applied traditionally in numerous DOMS models, indicating that these models do not simulate valid and realistic sports conditions [16,17]. Instead, during athletic activities such as running, change of directions, or jumps, eccentric contractions are shorter and part of the entire stretch-shortening cycle [9,30]. Remarkably, particularly for high-speed plyometric conditions, there is evidence to suggest that compression garments may function through a biomechanical support of the muscle-tendon unit, resulting in greater mechanical efficiency and promoting a lower energy expenditure [31,32] and reduced microtrauma and muscular damage [13]. From a biomechanical perspective, it has been further postulated that compression is able to reduce muscle vibrations and oscillations during repetitive landing loads, making plyometric contractions susceptible to compression [33]. However, only few studies have investigated real-time effects of compression on mechanical variables.

Heterogeneous strategies are discussed for the ideal form of compression. The manifestation of overexertion muscle injuries and DOMS is due to a complex sequence of local and systemic physiological responses [34,35]. The earliest clinical manifestations of DOMS commonly begin 6–12 h post-exercise and increase progressively in the context of an inflammatory response until reaching a peak pain level 48–72 h after exercise. The inflammatory process is associated with electrolyte imbalances, leukocyte accumulation, and an upregulation of circulating proinflammatory cytokines, accompanied by intramuscular edema, compartment swelling, nociceptor activation, and/or pain sensation. It is thought that applying compression generates an external pressure gradient that attenuates changes in osmotic regulation and reduces the spread of edema and swelling [11]. The majority of studies include measurement time-points up to 24–48 h after exercise [9,10,11,12]. During this period of time, electrolyte imbalances, leukocyte accumulation and an upregulation of circulating proinflammatory cytokines have been found in the context of DOMS. The released cytokines have attributed to be responsible for increased vascular permeability and microcirculation disturbances leading to interstitial fluid accumulation, intramuscular edema, and compartment swelling [34,35]. It is unclear if compression interventions during the immediate post-exercise period or early inflammatory phase (representative by a wearing period of 6 h) have an impact on the complex inflammatory response and the development of DOMS, respectively.

Based on pathophysiological foundations, studies on compression focus on different time-dependent application modes, including “during exercise” (i.e., biomechanical support) and “post-exercise” treatment aiming to limit the inflammatory processes mentioned above and thereby preventing the development and clinical signs of DOMS [2]. A meta-analysis found that several studies described recovery-enhancing effects for wearing compression garments both during exercise and afterwards, whereas other studies were not able to confirm these findings [28]. Most of the scientific studies reporting benefits of post-exercise compression recommend compression periods between 24 and 72 h after exercise, which may not be routinely applicable due to discomfort caused by the compression garments. Besides discomfort leading, e.g., to disrupted sleep wearing conditions of 24–72 h may not be transferable to elite athletes due to an interaction with different other recovery interventions such as physical therapy or thermal therapy. Our study aimed to address sports-specific conditions in both the exercise protocol and the wearing regime of compression garments, which was supposed to represent an ordinary day of intense training or competition: The continuous application during exercise and during the post-exercise period of exemplary 6 h (i.e., during the return trip after competition or in the evening after training) is practiced by many sports teams and athletes. The results presented elucidate a tendency toward an exposure time-dependent effect of compression for the post-exercise period after exhausting eccentric and plyometric exercises.

While our study increased the existing knowledge on compression in sports, some limitations should be mentioned. We acknowledge that our measurements may not be transferable to real sport-specific conditions, as a standardized exercise sequence involving eccentric and plyometric exercises does not mimic realistic sports-specific conditions. The preceding exercise intervention allows us to relate data to standardized exhausting muscle activity, an integral part of many sports. The chosen protocol has to be considered as effective as DOMS was induced in every participant. However, we were not able to investigate effects of compression on sporting activities in which different metabolic demands interact and may influence physiological responses continuously. Further, we used each subject as their own control. We chose an intraindividual control group because interindividual differences in DOMS expression have occurred in our preliminary tests and have been described in several studies [36,37]. However, one calf may influence the outcome of the other calf due to a systemic inflammatory healing response. Nevertheless, the findings may be significant for the practical implementation of compression garments. This study contributes to the ongoing controversial debate about when and how long compression should be applied. Hence, the remarkable findings obtained with this multiparametric approach can be valuable for further investigations. Longitudinal data assessments including multiple and frequent multiparametric measures during an observational period up to 72 h must be implemented in order to determine exact application times supporting recovery-enhancing effects in DOMS.

## 4. Materials and Methods

Study design: At baseline (T0), 6 h (T1), and 48 h (T2) after a standardized exercise protocol, MRI, creatine kinase (CK) levels, jump height, calf circumference, ankle dorsiflexion (DF), and muscle soreness were assessed. After the baseline measurements, an exercise intervention (described below) was performed with participants wearing a compression garment (21–22 mmHg, 75% polyamide, 25% elastane) on a randomly selected calf. Compression garments were worn continuously during the exercise intervention and for the following 6 h. The garments were removed immediately before the beginning of the second measurements (T1).

Participant recruitment and study population: Healthy individuals between 20 and 30 years of age were recruited from medical and sports facilities. Eighteen healthy volunteers (8 women and 10 men; mean age: 24.1 ± 3.6; range, 18–29 years, BMI 22.7 ± 2.7 kg/m^2^) were included in this study. Inclusion criteria were a lack of signs, symptoms, or history of chronic disease; no current acute or overuse injuries of the lower limb; and no history of muscle injury. All volunteers were asked to forego alcohol and sports for 72 h before and 48 h after the first MRI acquisition. Exclusion criteria were any symptoms of lower-limb muscle soreness in the 3 months prior to the study and regular training habits that included eccentric or plyometric exercises.

Ethical approval: The study was approved by the local institutional Ethics Committee and written informed consent was obtained from all volunteers (Ref. No. 33_16 B; Friedrich-Alexander-Universität (FAU), Erlangen-Nürnberg, Germany).

**Figure 3:**
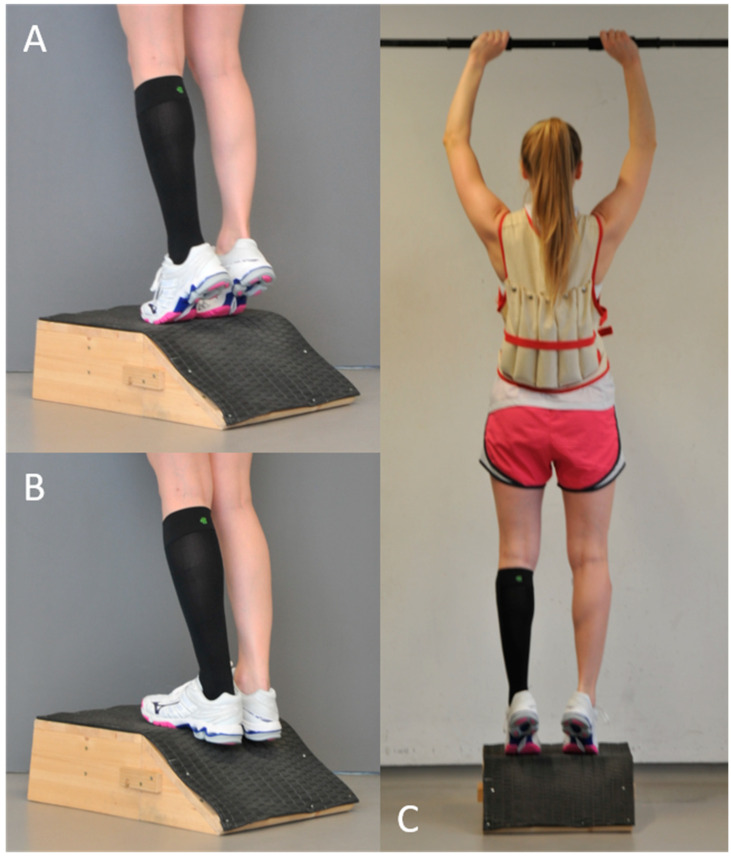
Procedure of the standardized eccentric exercise of the calf muscles: (**A**) start position, with heel raised; (**B**) eccentric contraction (−35°); and (**C**) composites of the entire setting.

Exercise intervention: All volunteers performed an established standardized training that included plyometric and eccentric exercises of both lower legs while wearing a compression garment on a randomly selected calf (left or right side). All exercises were monitored by an experienced strength and conditioning coach. Each participant warmed up using two sets of heel raises (15 repetitions per set), with a 20 s break between sets. Immediately after the warm-up, the plyometric exercise was performed. Volunteers completed 5 sets of 10 repetitions each of drop jumps from a 0.25 m box (5 s between jumps and 1 min between sets). Volunteers were asked to hop off from the box with both feet, land with both feet together, and perform a maximal vertical jump with the shortest possible contact time to the ground. After a 2 min break, a previously described eccentric exercise was performed on a specifically manufactured slant plate (−35°) [17,18]. To increase the load, each volunteer wore a weighted vest bearing approximately 40% of his/her body weight during the entire eccentric exercise (Figure 3). All participants performed 5 sets of 50 repetitions each and rested 60 s between sets, with the last set being performed until muscle fatigue, so that no further repetition of eccentric exercise was possible.

Imaging: MRI was performed on a 3T scanner (Magnetom Prisma; Siemens Healthineers; Erlangen, Germany) with an 18-channel body array coil. An axial T2-weighted turbo inversion recovery magnitude (TIRM) sequence (total acquisition time (AT), 4:53 min; inversion time (IT), 260 ms; echo time (TE), 69 ms; repetition time (TR), 5120 ms; resolution, 0.8 mm × 0.8 mm × 4.0 mm) and a coronal T2-weighted TIRM sequence (AT, 4:14 min; IT, 260 ms; TE, 68 ms; TR, 5870 ms; resolution, 0.9 mm × 0.9 mm × 4.0 mm) of both lower legs were acquired. An additional axial T2 mapping (AT, 9:35 min; TE, 10.6–190.8 ms; TR, 3170 ms; resolution, 0.7 mm × 0.7 mm × 8.0 mm) was applied to quantify the tissue water content. In addition, we performed a T1-weighted turbo spin-echo sequence to depict the anatomy and morphology of the lower leg.

To analyze the extent of edema, the T2 signal intensity and T2 time value (ms) were assessed according to previously published investigations [19]. The compartments of the lower leg were differentiated by defining specific regions of interest in the T1-weighted images corresponding to the anatomic margin of the medial (MGM) and lateral (LGM) head of the gastrocnemius muscle, the soleus muscle (SM), and the tibialis anterior (TA) muscle and copying these over the T2-weighted TIRM and T2 mapping sequence images. In addition, the volume of total intramuscular edema in the MGM was quantified using a threshold-based manual segmentation [19].

For grading of the exercise-induced muscle damage, a modification of the Peetrons classification [14] was used: grade 0 indicated a negative MRI without any visible pathology, grade I indicated edema but no architectural disorganization, grade II indicated architectural disruption indicating partial tear, and grade III indicated total muscle rupture. All subjects with grade I lesions in axial TIRM images were considered to have induced DOMS. The MRI images were analyzed blindly by an experienced radiologist using a professional image-processing program (syngo.via VB10; Siemens Healthineers; Erlangen, Germany).

Jump height: The power of both lower legs was assessed separately by jumps with full knee extension after familiarization with the movement pattern. Starting in an upright position with both arms fixed to the upper body and 90° flexion in the hip and knee of the contralateral leg, avoiding additional impulse movements, volunteers were asked to jump as quickly and explosively as possible in order to perform the highest possible jump. Tests were performed on a force platform (KMP Newton GmbH; Stein, Germany). The software provided by the manufacturer automatically calculates jumping height based on ground reaction forces. Mean jump height was determined using three repeated measurements.

Calf circumference: The maximum calf circumference was measured using a tape measure while the participant was sitting on the edge of a table with free-hanging lower legs [17]. The location of measurement was labeled with a permanent marker at baseline to ensure the same position for follow-up measurements.

Ankle dorsiflexion: Passive DF of both ankle joints was assessed manually with a goniometer while the participant was sitting on the edge of a table with free-hanging lower legs [38].

Muscle soreness: Self-reported muscle soreness was evaluated using a 10 cm visual analogue scale for pain (0, no pain; 10, worst pain). Pain scores were reported for each leg individually at rest and during activity (going down stairs) [17].

Creatine kinase (CK) levels: Blood CK levels were measured at baseline and at 6 h and 48 h after the eccentric exercise. Approximately 5 mL blood was collected by vein puncture from an antecubital vein into serum tubes. CK measurement was conducted using the UV test according to the IFCC method (37 °C; Cobas 6000; Roche Diagnostics; Mannheim, Germany).

Blinding: Test assistants and outcome assessors were kept unaware of which of the participants’ legs were supplied with compression garments and were not allowed to ask about this.

Statistical analysis: All assessed parameters were checked for normality with the Shapiro–Wilk test. In cases in which the data were normal, a repeated-measures analysis of variance, with time and treatment (compression versus non-compression) as the repeated independent variables, was performed for each of the dependent variables of interest: CK, T2 signal intensity, T2 time, calf circumference, jump height, and ankle DF. When the data were not normally distributed, a Friedman test with a post hoc Dunn–Bonferroni test was performed. Muscle soreness scores were analyzed using the Friedman test. All statistical tests were performed using SPSS Version 23 (IBM Corporation; Armonk, NY, USA), and *p* values of less than 0.05 were considered to indicate statistical significance.

## 5. Conclusions

This is the first study investigating the effects of wearing below-knee compression garments during exercise and a post-exercise period of 6 h on clinical, functional, and morphological outcomes in DOMS. The continuous application of below-knee compression garments during combined eccentric and plyometric exercises and for the following 6 h has no statistically significant effects on clinical signs of DOMS, jump performance, or the development of intramuscular edema observed in MRI.

## Figures and Tables

**Table 1:** Range of motion, circumference of the lower leg, jump height, T2 signal intensity, and T2 relaxation time within the medial head of the gastrocnemius muscle (MGM), total volume of intramuscular edema within the MGM, subjective pain at rest and while climbing stairs of the non-compressed and compressed lower leg before (baseline, T0) and 6 h (T1) and 48 h (T2) after plyometric and eccentric training. *p* values < 0.05 are bolded.

	Non-Compression					Compression					Non-Compression vs. Compression		
	Baseline (t0)	6 h (t1)	48 h (t2)	*p* Value (t0/t1)	*p* Value (t0/t2)	Baseline	6 h	48 h	*p* Value (t0/t1)	*p* Value (t0/t2)	*p* Value (t0/t0)	*p* Value (t1/t1)	*p* Value (t2/t2)
ROM (°)	38.0 ± 5.9	36.1 ± 7.9	33.2 ± 8.9	1.00	1.00	38.6 ± 6.8	37.4 ±8.7	32.4 ± 8.5	1.00	1.00	1.00	1.00	1.00
Circumference (cm)	36.9 ± 2.4	36.7 ± 2.4	37.3 ± 2.6	1.00	1.00	36.7 ± 2.4	36.4 ± 2.4	37.4 ± 2.5	1.00	0.182	1.00	0.693	1.00
Jump Height (cm)	8.1 ± 2.0	8.2 ± 2.3	6.8 ± 2.2	1.00	**0.027**	8.5 ± 2.2	8.0 ± 2.5	6.6 ± 1.7	1.00	**0.008**	1.00	1.00	1.00
T2 signal intensity	30.5 ± 10.2	36.7 ± 14.5	76.2 ± 49.3	1.00	**0.011**	28.5 ± 13.1	32.4 ± 13.4	83.1 ± 70.2	1.00	**0.005**	1.00	1.00	1.00
T2 relaxation time (ms)	36.3 ± 3.3	39.1 ± 5.8	54.9 ± 23.8	1.00	**0.007**	36.3 ± 3.9	38.4 ± 5.5	57.7 ± 27.9	1.00	**<0.001**	1.00	1.00	1.00
Volume of edema (mL)	12.1 ± 8.9	17.8 ± 11.0	106.4 ± 108.9	1.00	**0.003**	15.4 ± 13.0	18.3 ± 20.9	117.9 ± 135.0	1.00	**0.025**	1.00	1.00	1.00
Pain at rest	0.0 ± 0.0	0.4 ± 0.9	1.0 ± 1.5	1.00	0.223	0.0 ± 0.0	0.1 ± 0.3	0.7 ± 1.2	1.00	0.272	1.00	1.00	1.00
Pain at climbing stairs	0.0 ± 0.0	1.0 ± 1.1	4.8 ± 2.0	0.223	**<0.001**	0.0 ± 0.0	0.8 ± 0.9	4.7 ± 2.3	1.00	**<0.001**	1.00	1.00	1.00

## Data Availability

The data presented in this study are available on request from the corresponding author.
